# Stevens-Johnson Syndrome Following Vancomycin and Linezolid: A Real-World Analysis of Post-Marketing Surveillance Data

**DOI:** 10.3389/fphar.2022.872854

**Published:** 2022-04-28

**Authors:** Ming Ni, Xue-Dong Yin, Wen-Juan Hu, Na Zeng, Bin Zhao, Zhi-Ling Li

**Affiliations:** ^1^ Department of Pharmacy, Shanghai Children’s Hospital, Shanghai Jiao Tong University, Shanghai, China; ^2^ Department of Clinical Pharmacy, Shaoxing Maternity and Child Health Care Hospital, Shaoxing, China; ^3^ Shanghai Jiao Tong University School of Medicine, Shanghai, China; ^4^ Department of Pharmacy, Peking Union Medical College Hospital, Peking Union Medical College, Chinese Academy of Medical Sciences, Beijing, China

**Keywords:** vancomycin, linezolid, epidemiology, SJS, adverse event reporting system

## Abstract

**Background:** Stevens-Johnson syndrome (SJS) has been reported as a serious adverse effect in patients treated with vancomycin or linezolid, and there is currently a lack of real-world studies comparing specific differences in adverse effects of SJS.

**Methods:** According to the FDA’s Adverse Event Reporting System (FAERS), from January 2004 to July 2021, the data of suspected SJS after the use of vancomycin and linezolid were analyzed by imbalance and Bayesian analysis. The onset time, fatality rate and hospitalization rate of vancomycin-associated SJS and linezolid-associated SJS were also investigated.

**Results:** 276 cases of vancomycin-related SJS reports and 63 cases of linezolid-related SJS reports were identified. These two drugs are more common in middle-aged patients (45–64 years) than other age groups, and less common in underage children (<18). Among them, linezolid-related SJS is more common in middle-aged and elderly patients (45–74 years old) than other groups. Except for unspecified data, in vancomycin-associated SJS cases, there are more men than women (49.28% vs 43.84%), while in linezolid-associated SJS cases, the proportion of men and women is almost equal (44.44%). From the point of view of the areas where adverse reactions were reported, about 1/2 of the reports on Vancomycin-related SJS came from North America, and 1/3 of the reports came from Europe. The median onset time of Linezolid-related SJS was 5 days (interquartile range [IQR] 2–7.75), which was significantly earlier than that of Vancomycin-related SJS (12 days, IQR 4–20) (Mann-Whitney test, *p* < 0.0001). There were no significant differences in mortality and hospitalization rates after vancomycin and linezolid caused SJS.

**Conclusion:** The analysis of faers data provides a comprehensive overview of the adverse reactions of SJS caused by the use of vancomycin and linezolid, and can warn clinical workers to timely intervene and continuously monitor the patients at risk of SJS when using such drugs.

## Introduction

With the emergence of highly resistant β-lactam gram-positive bacteria, the use of vancomycin and linezolid has greatly increased (National Nosocomial Infections Surveillance System, 2004; Fridkin et al., 1999; Jones et al., 1999). Vancomycin was the first glycopeptide antibiotic to be introduced and is widely used in the treatment of MRSA and other Gram-positive bacteria. Linezolid is a new generation of fully synthetic oxazolidinone antibacterial drugs, which can be clinically used for the anti-infection treatment of pneumonia and vancomycin-resistant bacteria. The adverse reactions of vancomycin during use mainly include nephrotoxicity, ototoxicity and hematological toxicity. Therefore, vancomycin often needs to monitor the blood concentration during use, and adjust the dose to benefit while reducing the risk of adverse reactions. Common adverse reactions of linezolid are more prominent, such as thrombocytopenia, optic neuropathy, peripheral neuropathy, and lactic acidosis (Kishor et al., 2015).

With the increasing use of drugs, there are also concerns about serious adverse drug reactions. Drug eruptions are skin eruptions that are induced by drugs. Many drug eruptions belong to immunologically mediated reac-tions which are dose-independent and also termed type B adverse reactions. SJS is a potentially life-threatening immune-mediated adverse reaction characterized by extensive erythema, epidermal necrosis, and sloughing of the skin and mucosa. SJS is a rare but high burden disease. It is necessary to strengthen prevention, early diagnosis and long-term management (Chang et al., 2020).

The literature on vancomycin and linezolid-induced SJS is rare (Alexander and Greenberger, 1996; Jones et al., 2004; Minhas et al., 2016). Therefore, we can only obtain the association between vancomycin, linezolid and SJS through the reports in the faers database, which helps us to further study the differences between vancomycin related SJS and linezolid related SJS in time of onset, mortality and hospitalization.

## Methods

### Data Source

A retrospective pharmacovigilance study was conducted with data retrieved from January 2004 to July 2021 through the FAERS database. From the FAERS database, we selected reports of SJS caused by vancomycin or linezolid, and retrieved a total of 339 reports. Among them, there were 276 cases of vancomycin-related SJS reports and 63 cases of linezolid-related SJS reports, and duplicate data reports were deleted after referring to FDA recommendations.

### Adverse Event and Drug Identification

We investigated adverse events by using the MedDRA (Version 24.0) Preferred Terms as follows: epidermal necrosis (10059284), epidermal necrolysis (10014986), SJS (10042033), toxic epidermal necrolysis (10044223), Stevens Johnson reaction (10042029). Therefore, MICROMEDEX ^®^ (index nominum) was used like a dictionary. In addition, the trade names and common names of vancomycin and linezolid in the drug archives will also be listed.

### Data Mining

Based on the basic principles of Bayesian analysis and non proportional analysis, we applied reporting odds ratio (ROR), proportional reporting ratio (PRR), Bayesian confidence propagation neural network (BCPNN) and multiple gamma Poisson constrictor (MGPS) algorithms to explore the relationship between vancomycin or linezolid and SJS adverse reactions. The equations and standards of the four algorithms (DuMouchel, 1999; Evans et al., 2001; Szarfman et al., 2002; Van Puijenbroek et al., 2002; Hauben, 2003; Hauben et al., 2005; Norén et al., 2006; Ooba and Kubota, 2010; Szumilas, 2010) have been compiled into a table. See Table 1 for details. The extraction of these algorithms is mainly used to judge the tightness of the relationship between drugs and adverse events. As long as any one of the four algorithms meets the standard, it will be considered as a positive signal of SJS adverse events.

**TABLE 1:** Summary of major algorithms used for signal detection.

Algorithms	Equation^a^	Criteria
ROR	ROR=(a/b)/(c/d)	95% CI > 1, N ≥ 2
	${95\text{\%CI}=\text{e}}^{\text{ln}\left(\text{ROR}\right)\text{±}1\text{.}96\left(1\text{/a}+1\text{/b}+1\text{/c}+1\text{/d}\right)\text{\textasciicircum{}0}\text{.}5}$	
PRR	PRR=(a/(a+c))/(b/(b+d))	PRR≥2, χ^2^ ≥ 4, N ≥ 3
	$\chi^{2}=\sum \left({\left(\text{O}-\text{E}\right)}^{2}\text{/E}\right)\text{; }\left(\text{O}=\text{a, E}=\left(\text{a}+\text{b}\right)\left(\text{a}+\text{c}\right)\text{/}\left(\text{a}+\text{b}+\text{c}+\text{d}\right)\right)$	
BCPNN	${\text{IC}=\text{log}}_{2}\text{a}\left(\text{a}+\text{b}+\text{c}+\text{d}\right)\text{/}\left(\left(\text{a}+\text{c}\right)\left(\text{a}+\text{b}\right)\right)$	IC025 > 0
	${\text{IC}025=\text{e}}^{\text{ln}\left(\text{IC}\right)-1\text{.}96\left(1\text{/a}+1\text{/b}+1\text{/c}+1\text{/d}\right)\text{\textasciicircum{}0}\text{.}5}$	
MGPS	EBGM=a(a+b+c+d)/((a+c)(a+b))	EB05 ≥ 2, N > 0
	${\text{EB}05=\text{e}}^{\text{ln}\left(\text{EBGM}\right)-1\text{.}64\left(1\text{/a}+1\text{/b}+1\text{/c}+1\text{/d}\right)\text{\textasciicircum{}0}\text{.}5}$	

a a: number of reports containing both the suspect drug and the suspect adverse drug reaction. b: number of reports containing the suspect adverse drug reaction with other medications (except the drug of interest). c: number of reports containing the suspect drug with other adverse drug reactions (except the event of interest). d: number of reports containing other medications and other adverse drug reactions. Abbreviations: ROR, reporting odds ratio; CI, confidence interval; N, the number of co-occurrences; PRR, proportional reporting ratio; χ^2^, chi-squared; BCPNN, Bayesian confidence propagation neural network; IC, information component; IC025, the lower limit of the 95% two-sided CI, of the IC; MGPS, multi-item gamma Poisson shrinker; EBGM, empirical Bayesian geometric mean; EB05, the lower 90% one-sided CI of EBGM.

We separately counted the time to onset of SJS adverse reactions induced by vancomycin and linezolid, and recorded as the time interval between the start of drug administration and the occurrence of adverse events. Mortality data were reported as the ratio of reported deaths to vancomycin- or linezolid-related SJS reports. Also, all of the above reports are valid reports.

### Statistical Analysis

An applied descriptive analysis was made based on the clinical characteristics of patients with SJS induced by vancomycin and linezolid in the FAERS database. The time to onset of SJS induced by vancomycin and linezolid was compared using the Mann-Whitney test. Pearson’s chi-square test or Fisher’s exact test was utilized to compare the mortality and hospitalization rates between Vancomycin and Linezolid. The statistical significance was set at *p* < 0.001 with 95% confidence intervals. All statistical analyses were performed by the software GraphPad Prism 8 (GraphPad Software, CA, United States).

## Results

### Disproportionality Analysis and Bayesian Analysis

We searched the FARS database of 339 cases for all reports of vancomycin or linezolid-induced SJS from January 2004 to July 2021. Of these, 276 were SJS caused by vancomycin and 63 were SJS caused by linezolid. As shown in Table 2, according to the four algorithm criteria, vancomycin- and linezolid-induced SJS signals were detected, in which ROR, PRR, information component (IC), and empirical Bayesian geometric mean (EBGM) were all statistically significant.

**TABLE 2:** Signal detection for vancomycin-associated SJS and linezolid-associated SJS.

Drugs	N	ROR	PRR	IC	EBGM
		(95% two-sided CI)	(χ2)	(IC025)	(EBGM05)
Vancomycin	276	9.72 (8.62,10.96)*	9.56 (2097.18)*	3.24 (2.88)*	9.47 (8.57)*
Linezolid	63	3.46 (2.7,4.43)*	3.44 (109.19)*	1.78 (1.39)*	3.44 (2.79)*

ROR, reporting odds ratio; CI, confidence interval; PRR, proportional reporting ratio; χ^2^, chi-squared; IC, information component; EBGM, empirical Bayesian geometric mean.

### Descriptive Analysis

A summary of the clinical features reporting vancomycin- and linezolid-related SJS is presented in Table 3. With the exception of unspecified age, we found that SJS adverse reactions due to vancomycin and linezolid were more common in middle-aged patients (45–64 years) than in other age groups, minors (<18 years) are less common. Among them, the reported rate of linezolid-related SJS was more common in middle-aged and elderly patients (45–74) compared with other groups. In terms of patient gender, in vancomycin-related SJS cases, males were more likely to report than females (49.28% vs 43.84%), while in linezolid-related SJS cases, the proportion of males and females was almost Equal (29–28). In terms of regions reporting adverse reactions, approximately one-half of vancomycin-related SJ reports were from North America, and one-third were from Europe. Most reports on linezolid come from North America and Europe. Most reports are submitted by pharmacists, other health professionals and physicians, and reporting adverse drug events is the responsibility and obligation of every medical professional. Among them, pharmacists reported the most cases of vancomycin (27.9%) and doctors reported the most cases of linezolid (33.33%).

**TABLE 3:** Clinical characteristics of patients with vancomycin-associated SJS and Linezolid-associated SJS collected from the FAERS database (January 2004 to July 2021).

Characteristics	Reports (N, %)	
	Vancomycin	Linezolid
Patient age (year)		
<18	17 (6.16)	1 (1.59)
18–44	41 (14.86)	2 (3.17)
45–64	87 (31.52)	23 (36.51)
65–74	55 (19.93)	17 (26.98)
>74	41 (14.86)	4 (6.35)
Unknown	35 (12.68)	16 (25.40)
Patient gender		
Female	121 (43.84)	29 (46.03)
Male	136 (49.28)	28 (44.44)
Unknown	19 (0.36)	6 (1.59)
Area		
Africa	0 (0.00)	0 (0.00)
Asia	26 (9.42)	8 (12.70)
Europe	90 (32.61)	26 (41.27)
Oceania	0 (0.00)	0 (0.00)
North America	130 (47.10)	26 (41.27)
South America	6 (2.17)	1 (1.59)
Unknown	24 (8.70)	2 (3.17)
Reporters		
Consumer	7 (2.54)	3 (4.76)
Lawyer	11 (3.99)	0 (0.00)
Pharmacist	77 (27.90)	19 (30.16)
Physician	68 (24.64)	10 (15.87)
Other health-professional	66 (23.91)	21 (33.33)
Unknown	47 (17.03)	10 (15.87)
Outcome event		
Death	93 (33.70)	25 (39.68)
Disability	18 (6.52)	3 (4.76)
Hospitalization-Initial or Prolonged	144 (52.17)	28 (44.44)
Life-Threatening	59 (21.38)	17 (26.98)
Other Serious (Important Medical Event)	122 (44.20)	35 (55.56)
Required Intervention to Prevent Permanent Impairment/Damage	17 (6.16)	1 (1.59)

FAERS, FDA, adverse event reporting system.

### Time to Onset of Vancomycin- and Linezolid-Associated SJS

**FIGURE 1:**
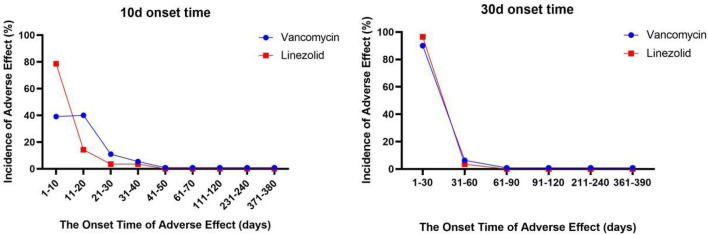
The onset time of vancomycin- and linezolid-associated SJS.

We describe the time to onset of vancomycin and linezolid in Figure 1. The median time to onset of linezolid-related SJS was 5 days (interquartile range [IQR] 2–7.75), significantly earlier than vancomycin-related SJS (12 days, IQR 4–20) (Mann-Whitney test, *p* < 0.0001). In general, vancomycin and linezolid-induced SJS had a higher onset time overall, while vancomycin and linezolid were special-use antibacterial drugs and should not be used for a long time. During use, more attention should be paid to monitoring adverse reactions at the initial stage of medication, especially linezolid.

### Fatality and Hospitalization Due to Vancomycin- and Linezolid-Associated SJS

To assess the mortality and hospitalization rates of SJS caused by vancomycin and linezolid to analyze the prognosis of SJS associated with vancomycin and linezolid. According to the report analysis, the hospitalization rate of vancomycin-related SJS was 52.17%, and the hospitalization rate of linezolid-related SJS was 44.44%. Unfortunately, vancomycin and linezolid did not differ significantly in mortality (33.7% VS 39.68%) and hospitalization after SJS.

The left figure only shows the change in the incidence of SJS every 10 days after administration for 380 days. The graph on the right shows the change in the incidence of SJS every 30 days after administration for 390 days.

## Discussion

As of now, this study is the first and largest report based on the FAES pharmacovigilance database from January 2004 to July 2021 to describe vancomycin- and linezolid-induced SJS in different vulnerable populations, onset time and adverse outcomes in real-world practice. The sample size of previous related studies is small, and most of them are single cases (Alexander and Greenberger, 1996; Waldman et al., 2004; Craycraft et al., 2005; Nasr et al., 2014), among which the literature reports of vancomycin causing SJS are more than that of linezolid. This article discusses the characteristics of SJS caused by vancomycin and linezolid from multiple perspectives, including mortality, hospitalization rate, and time to adverse reactions.

In the results of this study, we found that vancomycin-induced SJS is more common in middle-aged and elderly people. The population of case reports we collected in the PubMed database is also mostly middle-aged and elderly (Laurencin et al., 1992; Waldman et al., 2004; Craycraft et al., 2005; Craycraft et al., 2005). There are great differences in the pharmacokinetic behavior of vancomycin in different groups. After entering the human body, vancomycin is mainly metabolized through the kidney. Many factors may affect the pharmacokinetic behavior of vancomycin in the body, such as age, obesity, combined diseases, receiving other treatments and so on. There is evidence that infection is a serious cause of death in the elderly population over the age of 65 (Centers for Disease Control and Prevention, 2013). The risk of colonization and infection with methicillin-resistant *Staphylococcus aureus* (MRSA) in elderly patients is five times higher than in younger patients, resulting in a substantial increase in the frequency of vancomycin use (Barber et al., 2016). Changes in the physiological conditions of elderly patients are also potential factors that induce adverse drug reactions.

When SJS occurs, it is usually accompanied by eye diseases, and ophthalmologists need to intervene as soon as possible and follow up in a timely manner. The mechanism by which vancomycin and linezolid cause SJS is still unclear, mainly the immune pathogenesis, thus hindering the prevention and treatment of the disease. T cells may be the key mediators that induce SJS. Drugs, considered as foreign antigens, likely interact with particular HLA/peptide/T-cell receptor (TCR) complexes on keratinocytes to trigger the adaptive immune response and adverse reactions (Chang et al., 2020). The risk of SJS varies significantly by race or ethnicity with respect to the drugs used. It is not difficult to see from our research that North America and Europe have the highest incidence of SJS. We speculate that this may be related to the higher use of vancomycin and linezolid in North America and Europe. The more frequently antibiotics are used, the higher the resistance rate.

The global clinical and financial burden of SJS/ten is quite large, and the mortality rate of the elderly is even as high as 50% (White et al., 2018). In this study, hospitalization and mortality were also high after drug-induced SJS. The adverse outcomes of vancomycin-related SJS led to a hospitalization rate of 52.17%, and linezolid-related SJS led to a hospitalization rate of 44.44%, which was similar to mortality (44.44% vs 39.68%). Due to the low number of reported SJS associated with linezolid, we did not expect a significant difference between vancomycin and linezolid. The data can only show that once SJS is induced, both will lead to an increase in hospitalization rate and mortality, and the prognosis of patients is not ideal. However, we believe that early prediction and diagnosis will be more helpful for subsequent treatment.

Based on the FAERS database, the time to onsets of SJS for vancomycin and linezolid mainly occurred within 1 month after administration (vancomycin: median 12 days, IQR 4–20; linezolid: median 5 days, IQR 2–7.75), but there was a significant difference in average time to onset of SJS among vancomycin and linezolid (Mann-Whitney test, *p* < 0.0001). It can be seen that the time of SJS in patients using linezolid will be earlier than that of vancomycin. This requires our physicians and pharmacists to pay early attention to the occurrence of serious adverse skin reactions, especially SJS, when using linezolid. For vancomycin, the attention of physicians and pharmacists is required after 1 week of use.

The data in this study are all from professionals, including Pharmacist, Physician and Other health-professionals. This study fully demonstrates the advantages of real-world research and data mining technology, but there are also some limitations. Firstly, in the process of data mining, there will be incomplete information, such as input errors, report lack of important information and so on. These situations may lead to deviation in report analysis, which is an inevitable limitation of faers database. Secondly, we need to remove some duplicate or overlapping reports. When we try to delete some duplicate data based on event_dt, age, sex and reporter_country, a small part of the report may be lost. Due to the limited time, we can’t check all the report information one by one, so we can only eliminate the reports lacking information. Of course, our method needs further consideration. Third, confounding factors are difficult to control. The patient may already have a potential immune disease, which may aggravate the incidence of skin adverse reactions such as SJS. Fourth, disproportionate measurements lack incidence denominators, suffer from severe reporting bias, and are not subject to confounding adjustment (Michel et al., 2017; Raschi et al., 2018). Therefore, pharmacovigilance (analysis of spontaneous reporting system) cannot provide safety comparison or evaluate the association between drugs, especially the incidence of adverse events. The assumptions generated by disproportionate analysis also need to be further verified by more reliable and accurate methods. Although the above shortcomings do exist, the faers database can still help us identify the signals of vancomycin or linezolid and SJS. Our research may provide some help to the clinic for vancomycin and linezolid in the incidence of SJS.

## Conclusion

In his study, the signal of SJS after Vancomycin and Linezolid in real-world practice were determined according to the analysis of FAERS database. Compared with vancomycin, linezolid is less prone to skin rash and less prone to SJS. This may be related to the frequency of drug use, or it may be related to the fact that the drug itself is not easy to induce immune-related adverse reactions. However, the sample size of this study is limited, and more scientific researchers are required to work together to advance science and transform it into prediction, prevention, early diagnosis and treatment. Our study lays the foundation for a pharmacovigilance investigation, and further clinical pharmacy and pharmacoepidemiology may be required to test the hypotheses generated by this study for a more precise reporting analysis.

## Data Availability

The original contributions presented in the study are included in the article/Supplementary Material, further inquiries can be directed to the corresponding authors.
